# The Medical Association’s Meeting at Macon

**Published:** 1897-05

**Authors:** 


### EDITORIAL.

The office of the Business Manager of THE JOURNAL is 311 and 312 Fitten Building.

The editors are not responsible for views expressed by contributors.

Articles for publication should reach this office not later than the 15th inst.

Address all communications, and make all remittances payable to THE ATLANTA MEDICAL AND SURGICAL JOURNAL, Atlanta, Ga.

Reprints of articles will be furnished, when desired, at a small cost. Requests for the same should always be made *on the manuscript*.

### THE MEDICAL ASSOCIATION'S MEETING IN MACON.

This meeting was held on the 21st, 22d and 23d of April, and was one of the most successful and largely attended in the history of the Association.

Perhaps the greatest number of new members ever admitted joined at this meeting, there being a list of one hundred and one. One applicant was refused admittance because of some defect in his application, and one because it was reported that he was an eclectic. One honorary member was dropped on account of unprofessional nduct. The Board of Censors had several physicians explain hy their pictures had been in the papers and certain interviews. The plea was that it was done without their knowledge or consent.

A reception was tendered by Dr. K. P. Moore 6–8 P. M., a musicale at the Blind Academy 8–9, and a reception at the Cherokee Club 9 to ——, of the 21st. (The stiff punch at the latter is responsible for the dash in the preceding sentence.) At the club reception were many beautiful women; and the dancing, conversation and refreshments combined rendered it a most successful and enjoyable function. During the evening of the 22d a nice barbecue was served at Ocmulgee Park.

In all these affairs Macon did herself proud.

The large program showed many interesting and scientific papers, the reading and discussion of which were most instructive.

Dr. Lloyd, of New York, was there, genial as ever. His exhibition of X-ray work was highly enjoyed by a large audience of members and citizens, among them many ladies.

The distinguished Hunter McGuire read a paper on Appendicitis, which was a valuable contribution to the literature of the subject. His impromptu remarks soon after his arrival were most eloquent and touching.

This meeting passed without the, almost usual, "sensation," nothing worthy of comment in the papers having occurred.

Much credit is due to the President and the Committees for the success of the meeting.

The place of meeting for 1898 is Cumberland Island. Though Cumberland is far away from many of the members, we hope they will go, and "keep up the lick" they have begun. H.

---

THE "Jubilee Meeting" of the American Medical Association will be held in Philadelphia June 1st to 4th. A large attendance is expected. The committee of arrangements has set aside an hour on the second day of the meeting for exercises to commemo . rate the founding of the Association in Philadelphia in 1847. Dr N. S. Davis, who is recognized by all as the moving spirit in the enterprise, will read a short paper giving an account of the origin of the Association and how the objects for which it was founded have been attained. The committee has taken steps to secure the attendance at the meeting of the presidents of the State medical societies and the presidents of the State boards of medical examiners as an illustration of the success attained through the instrumentality of the Association.

In addition to the address of Dr. Davis, there will be two or three other short addresses to add to the interest of the occasion.

It is desired that the presidents of all State Boards of Examiners and of all State Medical Societies meet Dr. Davis a few minutes before his address in order that they may escort him to the stage. In the event of the president of any one of these organizations not being able to attend the meeting, he is requested to send as an alternate one of the ex-presidents, in order that every State society and every Examining Board may be represented upon this notable occasion.

Of the original members of the Association there are still living Dr. N. S. Davis, of Chicago, Dr. Alfred Stillé, of Philadelphia, Dr. John B. Johnson, of St. Louis, and Dr. David F. Atwater, of Springfield, Massachusetts. The committee hopes that these gentlemen will be present to take part in the meeting.

This semi-centennial meeting bids fair to surpass, in the character of the entertainment, the scientific papers, and the number in attendance, any meeting which has heretofore been held. The committee in charge have been able to obtain large and roomy places of meeting for the general meetings and the section meetings, all within a single block and within very short walking distance or immediately adjacent to the largest and most comfortable of the Philadelphia hotels.

For the week preceding and following the meeting the Committee of Arrangements have also arranged for clinical courses, which will be open without charge to all physicians who may visit the city at that time. These courses cover every branch in medicine and its specialties, and will afford visitors the opportunity of seeing the active clinical work of all the prominent teachers of Philadelphia.

---

THE *Medical Record of Mississippi* is the name of a new journal that comes from Biloxi, of that State. It makes a good beginning in its initial number, and under the editorship of Dr. H. H. Haralson it will no doubt deserve and obtain success.

